# Transforming Endothelium with Platelet‐Rich Plasma in Engineered Microvessels

**DOI:** 10.1002/advs.201901725

**Published:** 2019-10-16

**Authors:** Ryan J. Nagao, Raluca Marcu, Yuliang Wang, Lu Wang, Chris Arakawa, Cole DeForest, Junmei Chen, José A. López, Ying Zheng

**Affiliations:** ^1^ Department of Bioengineering University of Washington Seattle WA 98195 USA; ^2^ Department of Computer Science and Engineering University of Washington Seattle WA 98195 USA; ^3^ Institute for Stem Cell and Regenerative Medicine University of Washington Seattle WA 98195 USA; ^4^ Department of Environmental and Occupational Health Sciences University of Washington Seattle WA 98105‐6099 USA; ^5^ Department of Chemical Engineering University of Washington Seattle WA 98195 USA; ^6^ Bloodworks Northwest Research Institute Seattle WA 98102 USA; ^7^ Department of Medicine University of Washington Seattle WA 98109 USA

**Keywords:** endothelium, engineered microvessels, permeability, platelets, PRP

## Abstract

Vascularization remains an obstacle when engineering complex tissues for regeneration and disease modeling. Although progress has been made in recreating 3D vascular structures, challenges exist in generating a mature, functional endothelium. It is demonstrated that perfusing engineered microvessels with platelet‐rich plasma, a critical homeostatic component in vivo that is often overlooked in vitro, substantially transforms the endothelium, both maturing endothelial cells and improving functionality in 24 h. Platelets readily adhered to the exposed collagen‐I substrate through small gaps within engineered vessels without forming thrombi. The adherent platelets improve barrier function, enhance endothelial glycolysis, reduce thrombogenicity, and enrich smooth muscle cell growth surrounding the endothelium. These findings demonstrate that platelets are essential to the function of endothelium during vascular maturation and remodeling. This study sheds light on a potential strategy to engineer stable, implantable vascular networks.

## Graphical abstract

Perfusion of platelet‐rich plasma (PRP) substantially transforms the endothelium in engineered microvessels in 24 h. PRP increases vessel diameter, improves barrier function, enhances endothelial glycolysis, reduces thrombogenicity, and enriches smooth muscle cell growth surrounding the endothelium. This work provides a potential strategy to improve vascular maturation and remodeling in engineered complex tissues for implantation and disease modeling.

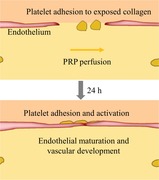

## 1 Introduction

Engineering complex tissues holds the promise of replacing damaged organs and establishing platforms to model disease and test drug candidates.^1^ Efforts have been made to incorporate perfusable vasculature to provide efficient blood supply in support of engineered tissue survival and growth.^2^ However, engineered vasculature has thus far not fully mimicked the function of native vasculature which has likely contributed to the limited growth and remodeling of tissues in vitro. One potential strategy for improving the function of engineered vasculature was suggested by studies performed over 50 years ago, when researchers attempted to increase tissue viability and vascular integrity in isolated perfused organs. Perfusion of platelet‐rich plasma (PRP) was found to preserve vascular integrity and prevent parenchymal inflammation compared to perfusion of plasma alone.^3^

Platelets are known for their critical role in preventing blood loss in response to injury, but their role in safeguarding the endothelium during homeostasis is often overlooked.^4^ This function is highlighted by the fact that patients with thrombocytopenia are at risk for spontaneous bleeding even without injury.^5^ Over the past 70 years, different roles for platelets have included forming physical plugs to exposed lamina,^3^, ^6^ elaborating bioactive vehicles that can improve endothelial barrier function by releasing soluble growth factors,^7^ and regulating transendothelial migration of leukocytes and thereby inflammation.[qv: 4b] Platelets may also have a role in vascular development, as remodeling of the capillary plexus coincides with the emergence of megakaryocytes^8^ and loss of CD41+ cells leads to animal lethality due to vascular defects.^9^ However, it is not known how the endothelium changes nor matures when interacting with platelets, nor whether platelets alone are sufficient to enhance vascular function. Better understanding of these questions would provide potential strategies to prevent vascular leakage and improve vascularization in engineered complex tissues.

Studies examining platelet–endothelium interactions have typically relied on either conventional 2D culture or animal models. In 2D transwell culture, transendothelial diffusion of albumin and other fluorescence molecules was reduced following treatment with platelets or platelet releasate, but not with fixed platelets.^7^ Removing the platelets reversed this effect, suggesting that growth factors released from unstimulated platelets were responsible. These early studies presumed that endothelial cells (ECs) did not physically interact with platelets and the presence of platelets only passively changed transendothelial diffusion. To date, few studies have sought to understand how the endothelium is influenced by platelets. Furthermore 2D systems lack a 3D architecture that allows for the formation of a continuous lumen that can be exposed to intra‐ and transluminal flow and continuous 3D stresses, all of which are integral components of vascular function. Alternatively, animal models have provided confounding observations when investigating the effect of platelets on the endothelium—demonstrating the ability of platelets to both decrease or increase vessel permeability in separate investigations.^10^ This is likely due to the complexity of in vivo systems, in which many contributing factors are coupled together. Thus, there is a need to study platelet–endothelium interactions in a system that better mimics the 3D vascular microenvironment in vivo while also allowing for the investigation of individual components in a stepwise approach.

In this study, we exploit an engineered 3D microvessel system to uncover PRP‐mediated vascular phenomena that have not been possible using 2D culture. This system comprises vessels within a collagen I matrix that allows for vascular remodeling in response to various types of stimuli: biophysical (hemodynamics), biochemical (angiogenic growth factors), and cellular, and has demonstrated unique utility in studies of angiogenesis and thrombosis.^11^ Using this system, we show that perfusing platelets induces substantial changes to vessel diameter, EC ultrastructure, and metabolism, as well as complex physiological processes such as barrier function, antithrombogenicity, and mural cell interactions. These effects were not evident in parallel 2D cultures nor using perfusates such as enriched plasma (EnP) or lyophilized platelets, suggesting endothelial maturation and maintenance requires metabolically active platelets in the context of 3D microvessels. Together, these findings help unveil the role of platelets in diverse vascular functions and provide a promising strategy to enhance the functionality of engineered vessels and complex tissues.

## 2 Results

### 2.1 PRP Treatment Alters Vascular Diameter and Barrier Functions in 3D Engineered Microvessels

**Figure 1:**
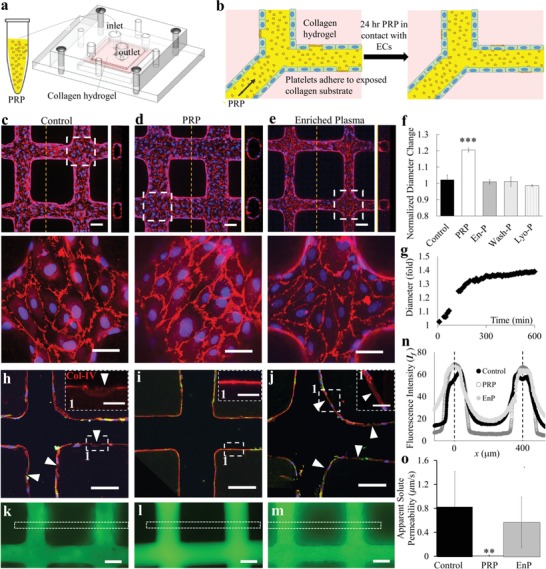
PRP perfusion improves 3D engineered vessel integrity and barrier function. a) Schematic of PRP perfusion into microvessels after 3 days of fabrication and b) zoomed diagrams of perfused PRP adhered to exposed collagen substrate and healed the endothelium. c–e) z‐Stack projection, cross‐sectional and enlarged views of representative confocal images of engineered vessels perfused with c) control media, d) PRP, and e) EnP. Red: CD31, blue: nuclei. f) Quantification of vessel diameter changes following perfusion with control media, PRP, EnP, washed platelets in Tyrode's buffer (Wash‐P), and lyophilized platelets in plasma (Lyo‐P). ***: *p* < 0.001. g) Time course of vessel diameter changes during PRP perfusion. h–j) Representative images of cryo‐sectioned microvessels following perfusion with h) control media, i) PRP, and j) EnP with enlarged views of dash boxes in insets. Red: Collagen IV, green: vWF, blue: nuclei. k–m) Fluorescence images after 3 min perfusion of 70 kDa FITC‐dextran through microvessels treated with k) control media, l) PRP, and m) EnP. n) Fluorescence intensity comparison across vessel network region (white dash boxes in (k–m)), with left vessel center set as *x* = 0. o) Quantification and comparison of apparent solute permeability for vessels in three conditions (control, PRP, and EnP). **: *p* < 0.01. Scale bars: c–e) 100 µm (50 µm in enlargements); h–j) 100 µm (25 µm in enlargements) k–m) 100 µm.

Engineered microvessels were fabricated in 7.5 mg mL^−1^ collagen I, seeded with human umbilical vein endothelial cells (HUVECs) and cultured under gravity‐driven flow for 3 days, as shown previously (Figure S1a, Supporting Information).^12^ At day 3 after endothelial seeding, each microvessel device was perfused with PRP, plasma that was incubated with platelets for 4 h prior to platelet removal—referred to here as EnP, or control media for 24 h and monitored for changes in vessel morphology and function (**Figure** **1**a,b). The 24 h time frame was chosen for PRP perfusion to eliminate the complications arising from platelet loss or apoptosis after prolonged storage at 37 °C. PRP treatment for 24 h caused vascular remodeling and increased vessel diameter, which reached an average of 120.4 ± 0.9% of the original diameter compared to negligible changes in control media (102.1 ± 2.2%) or EnP (100.9 ± 1.3%) (Figure 1c–f and Figure S1b, Supporting Information). This radial remodeling occurred isotropically (Figure 1b and Figure S1b, Supporting Information), suggesting the establishment of circumferential strain on vessel walls and therefore, increased vessel volume. When imaged in real‐time, vessel diameter increased linearly in the initial 2 h before gradually reaching a plateau for the remaining time (Figure 1g). We have noted that consistent increases in diameter occurred even after PRP treatment with platelet concentrations as low as 10% of physiological value (data not shown). We did not observe significant changes in vessel diameter when perfused with washed platelets resuspended in Tyrode's buffer or lyophilized platelets in EnP (101.0 ± 2.8% and 98.6 ± 0.6% of their original diameters, respectively) (Figure 1f and Figure S1b, Supporting Information). These data suggest that both platelets and plasma contribute to the increased vessel diameters of PRP‐treated vessels. ECs displayed similar junctions (i.e., VE‐Cad—not shown and CD31) at cell–cell contacts following treatment with PRP, EnP, and washed platelets (Figure 1d,e and Figure S1c, Supporting Information); however, junctions became disrupted when treated with lyophilized platelets in plasma (arrows and arrowheads, Figure S1d, Supporting Information).

We next examined changes to the vessel wall 24 h posttreatment by examining the deposition of basement membrane proteins and barrier function via immunofluorescence analyses. PRP‐treated microvessels had a thick and continuous layer of the basement membrane protein, Col‐IV, along their vessel walls, whereas control and EnP‐treated microvessels had regions of discontinuity (arrowheads, Figure 1h–j). Perfusion of fluorescently conjugated 70 kDa dextran solution in basal media enabled real‐time evaluation of fluorescence permeation from vessel lumen into the interstitium. Microvessels treated with EnP resulted in no significant effect compared to control with large and overlapping variations in both conditions, with measured permeability coefficient of (3.5 ± 2.3) × 10^−5^ cm s^−1^ in EnP versus (4.5 ± 3.5) × 10^−5^ cm s^−1^ in control. PRP treatment resulted in decreased fluorescent permeation regardless of the initial vessel conditions, demonstrating improved vessel barrier function compared to control and EnP (Figure 1k–m). The quantification revealed a 45‐fold reduction in apparent solute permeability ((9.9 ± 1.4) × 10^−7^ cm s^−1^) in PRP‐treated vessels with minimal variation compared to control microvessels (Figure 1n,o).

In contrast to engineered 3D microvessels, when PRP was applied to HUVECs cultured in 2D monolayers, we found many platelets adhered at regions between adjacent ECs as seen through phalloidin staining (Figure S2a, Supporting Information). These platelet–endothelial interactions appeared to have impaired the integrity of VE‐cadherin junctions between adjacent cells, resulting in EC retraction (Figure S2a‐ii,iii, Supporting Information). There was a significant decrease in the number of cells per surface area comparing control media (444 ± 10 cells per mm^2^) to that after PRP treatment for 1 h (403 ± 29 cells per mm^2^) and for 24 h (357 ± 10 cells per mm^2^) (Figure S2b, Supporting Information).

### 2.2 Platelets Can Activate and Form Plugs when a Minimum Endothelium Defect is Exceeded

**Figure 2:**
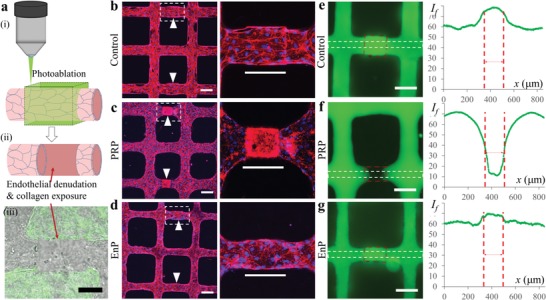
Engineered vessel ablation leads to occlusion after PRP treatment. a) Schematic of photoablation on vessel branches using two‐photon microscopy i) to create sites of EC denudation and collagen exposure (ii—schematic, and iii—overlay of brightfield and second harmonic generation images near ablation area). b–d) Confocal z‐stack images of vessels at 24 h following photoablation and perfusion with b) control media, c) PRP, and d) EnP. Right panels: enlarged views of dashed regions from the adjacent panel. Red: F‐actin, blue: nuclei. e–g) Fluorescent images of FITC‐dextran perfusion through the vessels for e) control, f) PRP treatment, and g) EnP treatment. Right panels: fluorescence intensity along the *x*‐axis for the corresponding dash boxes in the left panels. Red dash lines correspond to the ablated regions. Scale bars: a) 100 µm; b–d) 100 µm (150 µm in enlargements); e–g) 150 µm.

Platelets are classically believed to become activated following adhesion to exposed lamina then aggregate and recruit other platelets to form a plug during primary hemostasis. When perfused through normal engineered microvessels, some platelets adhered to microvessels but failed to form large aggregates (Figure S1, Supporting Information). To verify the activation status and aggregation capacity of the platelets in PRP, we created a band injury within individual microvessel branches (**Figure** **2**a). The endothelium in three vessel branches was photoablated to create 150 µm long hyperrectangular defects. The endothelium was removed in the photoablated regions along with small amount of basal collagen (Figure 2a). Within 30 min following photoablation, vessels were treated with PRP, EnP, or control media for 24 h (Figure 2b–d). In microvessels perfused with control media, ECs grew over the ablation sites, with increased vessel permeability in that region (Figure 2b,e). In the PRP‐treated microvessels, a thrombus formed that was confined to the injury site (Figure 2c). Regions outside the injury site exhibited consistent barrier enhancement and vessel enlargement as seen through fluorescently conjugated dextran perfusion (Figure 2f). No thrombus was observed with EnP perfusion and the endothelium grew over the ablation sites, with increased dextran permeability, similar to control (Figure 2d,g). When ablated regions were reduced to 10, 25, and 50 µm in diameter and 20 µm depth, cells in the ablated areas were able to heal completely following 24 h PRP treatment with no signs of thrombus formation (Figure S3, Supporting Information). Together these data suggest that platelets are normally not activated in PRP‐treated microvessels but can activate and form plugs when a minimum defect size is exceeded.

### 2.3 Platelets Adhere Abluminally and Cause EC Cytoskeletal Reorganization

**Figure 3:**
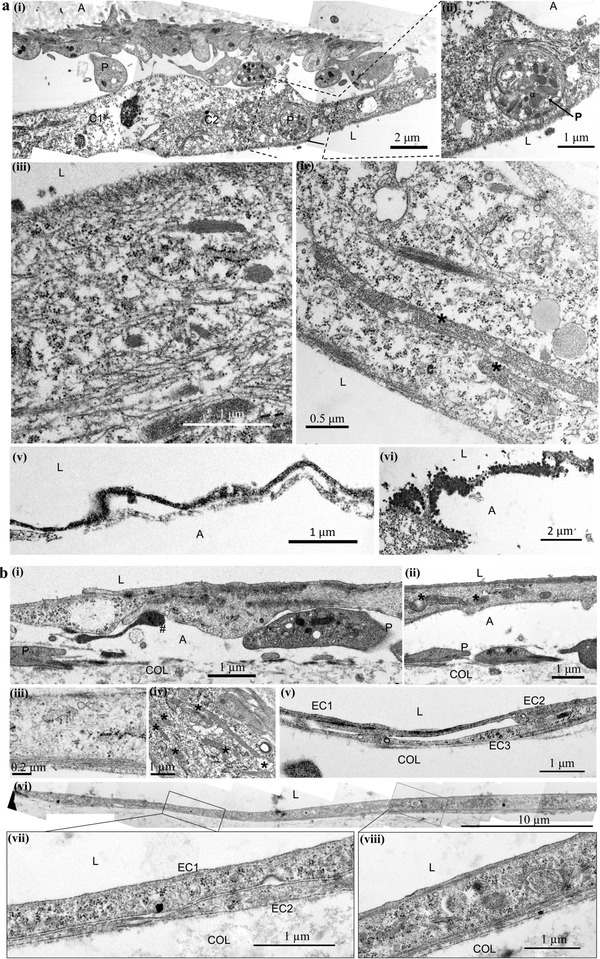
Ultrastructural analysis of engineered microvessels treated with PRP. a) Following 1 h PRP perfusion, platelet (labeled as P) distribution was only observed i) abluminally (labeled as A) or in some cases ii) internalized within ECs. iii,iv) Increased microtubule activation, mitochondrial (labeled with *) elongation, and fragmentation were visualized. v,vi) Electron dense material, appeared as dark granules, was found evenly distributed along the endothelium and in cell junctions. b) i,ii) After 24 h PRP perfusion, partial activation of platelets (P) could be distinguished through the loss of dense granules and presence of pseudopodia (#). iii,iv) Microtubule and mitochondrial (*) activation were still observed. v) Complex and vi–viii) overlapping cell junctions could be visualized. A: ablumen, L: lumen, P: platelets, C: cells, Col: collagen substrate.

We next examined the ultrastructural interactions of platelets and endothelium in 3D microvessels following PRP treatment using transmission electron microscopy (TEM). Pronounced ultrastructural changes occurred to ECs at different time points (i.e., 1 and 24 h) post PRP treatment which were not seen in vessels treated with EnP (Figure S4, Supporting Information). At 1 h following PRP treatment, the majority of platelets (P) were located on the abluminal side (A) of ECs without significant aggregation (**Figure** **3**a‐i). There were roughly two to four layers of platelets adhered, most of which lost their granules, likely during initial adhesion and partial activation, but maintained intact membranes. Some adherent platelets still contained numerous granules, and occasionally, rounded platelets rich in granules were also found internalized within ECs (Figure 3a‐ii). Increased actin polymerization and microtubules were seen throughout the cytosol of the ECs, with apparent cortical assembly (Figure 3a‐iii). Morphological changes were found in mitochondria, including elongation in excess of several micrometers and fragmentation of the membrane (asterisks, Figure 3a‐iv). In addition, an electron‐dense layer was formed on the luminal surface (L) of the endothelium (Figure 3a‐v) and appeared to bridge gaps between adjacent ECs (Figure 3a‐vi). At 24 h, intact platelets were again found on the abluminal surface of the endothelium (Figure 3b‐i). Cortical actin assembly was appeared to progress into a submembranous web within the endothelium (Figure 3b‐iii). Mitochondria showed extended length inside the cells, exceeding several micrometers (asterisks, Figure 3b‐iv). Adjacent ECs developed long overlapping junctions with lengths exceeding 30 µm (Figure 3b‐vi,vii). Overall, these ultrastructural data suggest that ECs underwent substantial structural reorganization and appeared under tension with partial platelet activation and minimal aggregation.

### 2.4 Molecular Signatures Demonstrate PRP Treatment Leads to Endothelial Maturation

**Figure 4:**
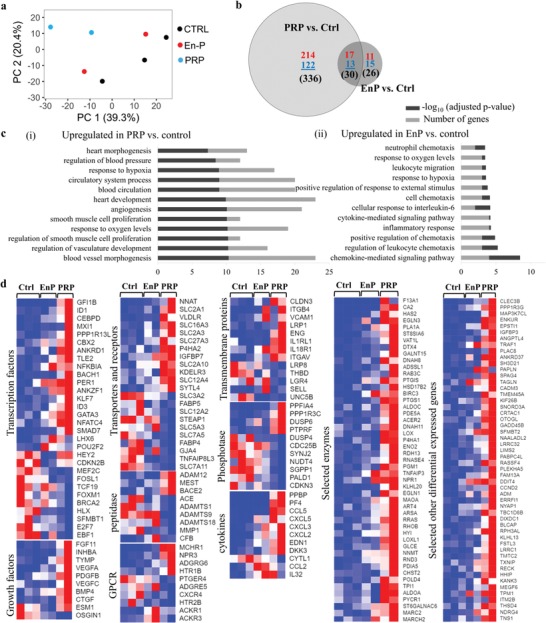
Transcriptional profiling of endothelium from engineered vessels after treatments with PRP or EnP compared with control. a) Principle component analysis for endothelium in three conditions (control, EnP, and PRP). b) Venn diagram showing differentially expressed genes comparing PRP versus control and EnP versus control. c) Top upregulated GO terms for PRP or EnP versus control. d) Heatmaps of selected genes for three conditions by category.

To gain better understanding of molecular characteristics of the endothelial response to PRP, we lysed engineered microvessels and collected RNA for transcription profiling following exposure to three conditions (*N* = 3, 2, 2 for control, PRP, and EnP conditions, respectively; each replicate was pooled from three microvessels at the same condition). Principle component analysis illustrated distinct clustering of PRP‐treated cells versus control, whereas EnP‐treated cells were clustered between the two but closer to the control (**Figure** **4**a). Differential expression analysis identified 366 genes differentially expressed with statistical significance in the PRP‐treated endothelium compared to the control, with 231 genes upregulated and 135 genes downregulated (fold change >1.5 and false discovery rate (FDR) < 0.05) (Figure 4b). Gene Ontology (GO) terminology analysis showed PRP‐treated vessels have significant upregulation of transcripts from genes involved in blood vessel morphogenesis, vascular development, regulation of smooth muscle cell (SMC) proliferation, response to oxygen levels, angiogenesis, and other vascular and circulatory system development (Figure 4c‐i). Canonical pathway analysis identified HIF1 signaling as the top upregulated pathway in PRP‐treated microvessels. In contrast, EnP‐treated vessels showed 56 differentially expressed genes with 28 upregulated compared to the control. Of these 28 genes, 17 were also upregulated in PRP treatment. GO analysis revealed that EnP‐treated microvessels had significant upregulation of pathways involved in chemokine and cytokine signaling, regulation of chemotaxis, and inflammatory responses, suggesting EnP‐stimulated microvessels with cytokines and chemokines (Figure 4c‐ii). This is likely due to the factors released from unstimulated platelets, as suggested in previous work.^7^

The top genes that were upregulated in PRP‐treated microvessels compared to control include transcription factors such as *SMAD7*, *PPARG*, *ID1/2/3*, *CEBPD*, *ANKRD1*, and *GATA3*, most of which are known to regulate angiogenesis and vascular development;^13^ transporters and receptors such as *SLC2A1*, *SLC16A3*, *SLC2A3*, *SLC27A3*, *SLC2A10*, *SLC12A4*, *VLDLR*; transmembrane proteins such as *CLDN3*, *ITGB4*, *VCAM1*, *ENG*, *LRP1*, are known to promote cell adhesion to cells and matrices; growth factors such as *FGF11*, *INHBA*, *TYMP*, *VEGFA*, *PDGFB*, *VEGFC*, *BMP4*, *CTGF*; cytokines such as *CXCL5*, *CXCL3*, *CXCL2*, *EDN1*, *DKK3*; and enzymes such as *HAS2*, *PLA1A*, *DTX4*, *PTGIS*, *PTGS1*, *ENO2*, *PGM1*, *NPR1*, *EGLN1*, *ART4*, *RRAS*, *RHOB*, are known to enhance vascular barrier function, vascular homeostasis, antiinflammatory, and antithrombotic functions^14^ (Figure 4d). Upstream regulator analysis in the PRP‐treated microvessels revealed increased activity in transcription regulators such as *HIF1A*, *STAT4*, *SMAD3*, *SMAD4*, *NOTCH1*, *EPAS1*, *STAT1*, *ARNT*, *CTNNB1*, *NURP1*, *HNF1B*, *GLI3*, *SMAD2*, *HMGA1*, *FOXO3*, *HAND1*, transmembrane receptors *IGF1R*, *TLR3*, *TLR7*, and F3; growth factors: *BMP4*, *AGT*, *TGFB1*, *TGFB3*, and *GDF9*; kinases: *TBK1*, *MAP3K8*, *TGFBR1*, *PTK2*, *BRD4*, *PIK3R1*, *CDK9*, *IKBKB*; growth factors such as *AGT*, *TGFB1*, *BMP4*, *TGFB3*, and *GDF9*; and cytokines such as *IFNG*, *TNF*, *IL1B*, *CSF1*, *EDN1*, and *CXCL12*. All these data suggest that PRP promoted vascular development and EC development at the molecular and transcriptional level.

### 2.5 PRP Shifts EC Metabolism from OXPHOS to Glycolysis

**Figure 5:**
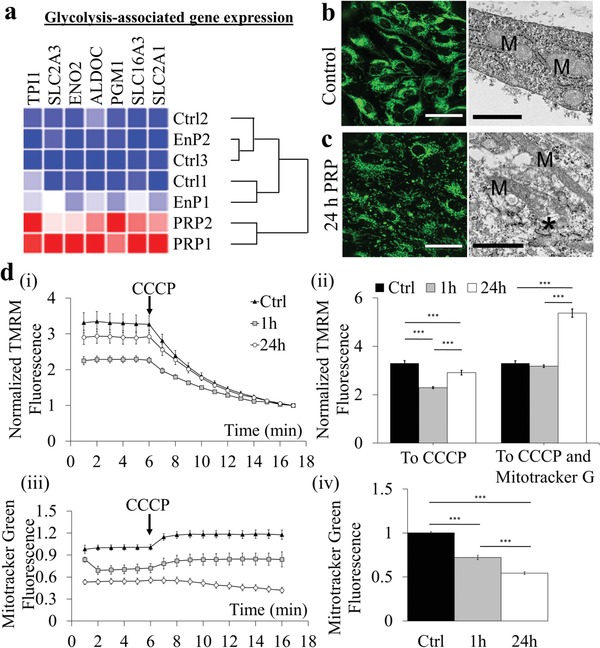
PRP treatment increases glycolytic activity in engineered vessels. a) Heatmap of selected glycolysis‐associated gene expression across conditions. b,c) MitoTracker Green staining of ECs in microvessels, b) control and c) after 24 h PRP treatment. Green: MitoTracker Green. Right panels: ultrastructural images showing mitochondrial (M) morphological elongation and fragmentation (*) after 24 h PRP treatment. Scale bars: b) 50 µm and c) 1 µm. d) Normalized TMRM fluorescence in microvessels over time comparing i) control media to treatment with PRP for 1 and 24 h; and ii) normalized to CCCP or CCCP and MitoTracker Green. iii,iv) MitoTracker Green fluorescence in microvessels (iii) over time comparing control, 1 h PRP, and 24 h PRP treatment. ***: *p* < 0.0001.

Mature endothelium in vivo relies heavily on glycolysis for adenosine triphosphate (ATP) production. We next examined metabolic activities via gene expression and mitochondrial function. Among differentially expressed genes, a subset related to metabolic activity was identified in PRP‐treated microvessels that suggested a shift from oxidative phosphorylation (OXPHOS) to glycolysis. In total, 34 genes associated with glycolysis were upregulated including 7 enzymes involved in glucose and lactate transport (*PGM1, TPI1, ALDOC, SLC2A3, SLC2A1, ENO2, SLC16A3*) that were selected for comparison (**Figure** **5**a). Euclidian distance hierarchical clustering demonstrated that PRP groups were distinct from control and EnP‐treated conditions (Figure 5a).

We then used MitoTracker green to directly visualize mitochondrial mass and structure via fluorescence imaging, which showed dense perinuclear mitochondrial networks in control microvessels (Figure 5b); these became punctate and fragmented at 24 h following PRP treatment (Figure 5c). This is consistent with the ultrastructural analysis, which showed elongation and membrane disruption (asterisks) of mitochondria (M) in endothelium after PRP treatment (Figure 3a‐iv,b‐iv). In comparison, the mitochondria in EnP‐treated microvessels incurred no such changes in mitochondria ultrastructure (Figure S4a, Supporting Information). These findings suggest a dynamic change to metabolic activity following PRP treatment with general trends in association with the reduction of mitochondrial mass and activity.

To measure OXPHOS activity within ECs after PRP treatment, we perfused tetramethylrhodamine methyl ester (TMRM) and MitoTracker green through microvessels at 1 and 24 h, and quantified mitochondrial membrane potential and total mitochondrial mass, respectively, using confocal fluorescence microscopy (Figure S5, Supporting Information). Fluorescence levels were normalized following the addition of the protonophore, carbonyl cyanide m‐chlorophenyl hydrazone (CCCP) to depolarize the mitochondrial membrane. The normalized membrane potential of mitochondria within ECs was diminished following PRP treatment (2.3 ± 0.42 and 2.9 ± 0.48 for 1 and 24 h treatments, respectively) compared to control (3.3 ± 0.97) (Figure 5d‐i,ii). The total mitochondrial mass normalized to control was also diminished following exposure to PRP, falling from 1.0 ± 0.17 in control vessels to 0.72 ± 0.26 and 0.54 ± 0.11 after 1 and 24 h PRP exposure, respectively (Figure 5d‐iii,iv). However, when normalized to total mitochondrial mass, the activity of remaining mitochondria in PRP‐treated microvessels was unaffected after 1 h (3.2 ± 0.58) and elevated after 24 h (5.4 ± 0.89) compared to control (3.3 ± 0.97) (Figure 5d‐ii), These results indicate that although total mitochondrial mass and membrane potential decreased, the mitochondria remaining following PRP perfusion were functioning at normal and elevated activity levels at 1 and 24 h. Together, reduced mitochondrial mass and membrane potential support the idea of reduced contribution of OXPHOS to support cellular energetic demand and a shift toward glycolysis.

### 2.6 PRP Enhances SMC Density around Engineered Vessels

**Figure 6:**
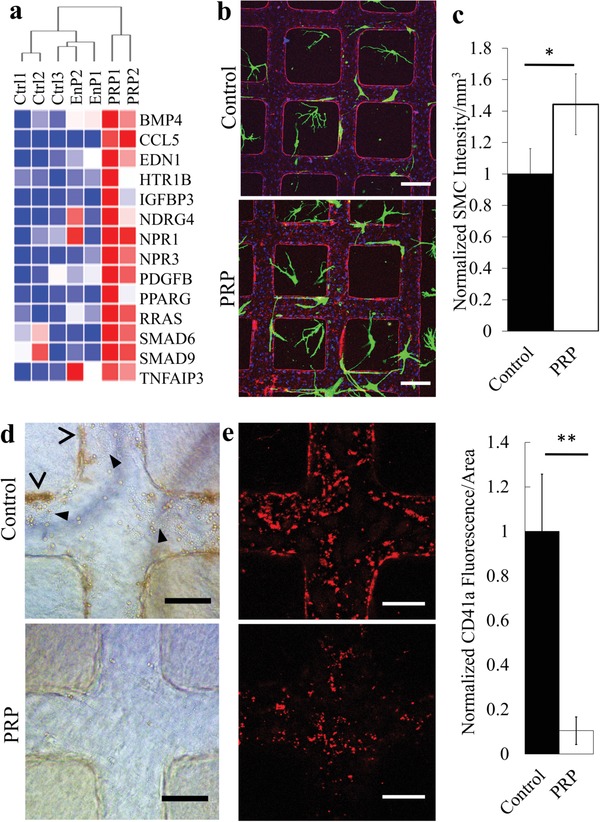
PRP treatment induces functional changes in engineered microvessels. a) Heatmap of selected gene expressions associated with regulation of SMC proliferation. b) Projection of z‐stack confocal images of microvessels in control (top) and PRP treated (bottom) vessels. Red: CD31, green: SMCs, and blue: nuclei. Scale bar: 200 µm. c) Quantification of SMC intensity, significantly increased following PRP treatment. *: *p* < 0.05. d,e) Microvessels after treatment with control media (top panels) and PRP (bottom) then perfused with whole blood for 30 min. Brightfield images d) reveal the presence of bound and extravasated red blood cells. e) Immunofluorescence images of CD41‐PE labeled platelets adherent on vessel walls following whole blood perfusion (left panels). Quantification of fluorescence intensity per area was significantly higher in control vessels compared to PRP‐treated (right panel). **: *p* < 0.01. Scale bars: d,e) 100 µm.

Another important component involved in the maturation and stabilization of the developing endothelium is mural cell support. PRP treatment appeared to upregulate the expression of EC genes involved in SMC proliferation (*CCL5, EDN1, IGFBP3, NPR3, BMP4, PPARG, TNFAIP3, PDGFB, HTR1B, NDRG4, NPR1*) as well as recruitment (*PDGFB, RRAS, SMAD6, SMAD9*) (**Figure** **6**a). To assess the functional ramifications of these gene changes, we incorporated green fluorescent protein (GFP)‐expressing coronary SMCs in the matrix surrounding our microvessels. These co‐cultured microvessels were maintained for 3 days prior to the addition of PRP or control medium for 24 h. Vascular patency remained throughout PRP perfusion for 24 h with no thrombus formation (Figure 6b). SMC density significantly increased in the PRP‐treated vessels compared to control vessels (1.44 ± 0.17‐fold) (Figure 6c). These findings suggest that PRP could potentially promote EC maturation via enhanced mural cell support.

### 2.7 PRP Improves Antithrombotic Potential of Engineered Microvessels

Healthy mature vasculature has antiadhesive and antithrombotic properties. We therefore examined the effect of PRP treatment on blood cell adhesion on engineered vessel walls with or without prior PRP treatment. Whole blood was perfused through the microvessels for 15 min with platelets labeled with CD41a‐PE and visualized in brightfield and immunofluorescence microscopy after washing and fixation. There were fewer adherent platelets (arrowheads) and red blood cells (RBCs), and fewer RBC extravasations and petechiae (chevrons) in PRP‐treated microvessels compared to control (Figure 6d). PRP‐treated microvessels had over a 90% reduction in platelet area coverage, from (8.0 ± 1.8)% in control to (0.8 ± 0.4)% (Figure 6e). CD41a‐PE platelets were also seen in larger aggregates in control than that on PRP‐treated microvessels. PRP‐treated vessels appeared to have abundant residual platelet material (positive for phalloidin and von Willebrand factor (vWF) staining but were negative for CD41a‐PE) (Figure S6, Supporting Information), suggesting these residual platelets were likely in the abluminal side of the endothelium, as seen in ultrastructure analyses.

## 3 Discussion

Over 50 years ago platelets were found to be vasculoprotective during ex vivo organ perfusion.^3^ Many subsequent studies have sought to understand the mechanism of this phenomenon, which falls outside the normal hemostatic function of platelets. Platelets contain many molecules capable of influencing other cells. With regard to endothelial function, some of these improve barrier function whereas others increase vessel permeability.^15^ Although platelets are of obvious importance to endothelial maintenance and functions, the mechanisms by which they carry out these activities have been hard to elucidate. Conventional 2D studies lack the characteristics required for normal physiological interactions between ECs and platelets, e.g., 3D luminal geometry, pressure and flow relationships, and appropriate cell–cell contacts. In this study, we used 3D engineered microvessels to demonstrate that PRP transformed the endothelium by improving vessel barrier function, expanding vessel diameter, thickening the abluminal lining, promoting glycolysis, and enhancing smooth muscle recruitment and antithrombogenicity. These observations are unique in 3D microvessels, whereas in 2D endothelial cultures, bound platelets were found to disrupt EC junctions, leading to cell retraction.

Normal plasma has high protein content, resulting in a high oncotic pressure gradient of approximately 30 cmH_2_O between our engineered microvessel lumen and the surrounding matrix, whereas the hydrostatic pressure gradient across the microvessel lumen is 1 cmH_2_O. Based on the Starling equation, the overall pressure drop would lead to rapid water reabsorption into the vessel, increasing the vessel volume and wall tension. This increase of vessel diameter over time suggests that platelets enhance vessel barrier and maintenance of the oncotic pressure drops across the vessel wall. The increased wall tension is also suggested in our ultrastructural analysis, which identified increased cytoskeleton polymerization in vessels after 1 h PRP perfusion and the formation of a submembranous actin web after 24 h PRP perfusion. The EnP‐treated microvessels, though having the same oncotic pressure difference, failed to induce these vessel wall changes. Furthermore, lyophilized platelets perfused in EnP also failed to induce these changes, suggesting physical interactions with metabolically active platelets are critical to induce vessel wall changes in this process. This substantial cytoskeletal remodeling could also be in response to the partial activation of the adhered platelets. Many of the factors released by platelets are associated with inflammatory responses and endothelial reorganization. The long‐term effects of these processes on endothelial homeostasis will require further investigation.

Platelets appear to interact with the vessel wall in a biphasic manner. During PRP perfusion in normal vessels, platelets failed to form large aggregates or vascular occlusions. Platelets were found covered and sometimes internalized by the endothelium as soon as 1 h after perfusion and maintained the same morphology after 24 h with no signs of further activation nor aggregation. In contrast, when a large region of the vessel was denuded of endothelium, platelets became locally activated and formed a plug to occlude a vessel branch. When the denuded region was reduced, no platelet plugs formed and the vessel did not become occluded. This suggests healthy endothelium provides negative cues to suppress platelet activation and aggregation.

PRP also promoted vascular maturation and improved functionality. Our transcriptional profiling of 3D engineered microvessels after PRP treatment showed significant upregulation of transcripts involved in vascular development and remodeling. Although genes associated with inflammation were upregulated, GO term and canonical pathway analysis suggest that PRP treatment predominantly affects vascular development and maturation instead of vascular activation. We focused on three aspects of vascular functions supported by corresponding gene regulation: metabolism, mural cell support, and thrombogenicity. Among the >300 differentially expressed genes after PRP treatment, 10% (over 30) were directly associated with glycolysis. The upregulation of transcripts involved in glycolysis coincided with decreased mitochondrial activity in the microvessels, which provide evidence of a shift in EC metabolic activity. In normal blood vessels, glycolysis accounts for over 85% of the total cellular ATP production in ECs,^16^ and mitochondrial activity remains low in mature vasculature. It remains unknown how this change in EC metabolism will affect long‐term tissue growth and remodeling in vitro or tissue survival after implantation in vivo. It is possible that vascular pre‐conditioning toward physiological conditions could promote better survival and integration once implanted.

Our data also showed PRP treatment upregulates transcripts in platelet‐derived growth factor signaling and increases mural cell density around microvessels, suggesting platelets could be contributing to vascular development. Though the direct coating of endothelium by mural cells was not obvious, the increase of mural cell density suggests effective paracrine signaling after 24 h PRP treatment. Future long‐term studies could facilitate our understanding of the role of platelets in mural cell recruitment and continued vascular development. The decreased platelet binding and blood cell interactions after PRP treatment suggest that PRP could further serve as a pre‐conditioning procedure to reduce vascular thrombogenicity prior to anastomosis in vivo.

Overall, our work demonstrates that PRP transforms the endothelium into mature and functional vessels. Although the exact mechanisms of this transformation are yet to be discovered, our work opens up new opportunities to study the role of blood components in the development of a functional vasculature and potentially in engineering complex and functional tissue.

## 4 Experimental Section

Unless otherwise stated, all materials were purchased from Sigma Aldrich. Fresh blood was drawn from consenting healthy donors, who were nonsmokers and drug and aspirin free for at least 3 days prior to blood donation, under protocols approved by the Institutional Review Board of the University of Washington.

*Perfusate Preparation*: PRP were obtained by fractionating 3.2% sodium citrate‐treated whole blood at 200 *g* for 15 min with no brake. The PRP portion was then moved to a separate tube with a transfer pipet and allowed to rest for several hours prior to use. For EnP, PRP was centrifuged at 1200 *g* for 10 min, the supernatant was collected and centrifuged again at 1200 *g* for 10 min. The supernatant collected after second spin was gently filtered through a 0.2 µm cellulose membrane to further eliminate platelets from the plasma. EnP was selected as a control to PRP given the presence of soluble growth factors that had been used to decrease EC permeability in 2D monolayers previously, as well as equivalent oncotic pressures resulting from the proteins within plasma.^6^ Lyophilized platelet solution was made by adding lyophilized platelets (Bio/Data Corp., Horsham, PA) to the EnP at 2.5 × 10^8^ mL^−1^.

Washed platelets were obtained from whole blood in ACD (trisodium citrate (13.2 g L^−1^), citric acid (4.8 g L^−1^), and dextrose (14.7 g L^−1^)) (1:6–1:8) vol:vol from normal volunteers who were nonsmokers and drug and aspirin free for at least 3 days prior to blood donation. Blood was centrifuged at 120 *g* for 15 min with no brake. PRP was transferred to a separate tube and centrifuged at 1200 *g* for 10 min. The plasma supernatant was discarded and the platelet pellet was resuspended in CGS buffer (sodium citrate (3.8 g L^−1^), glucose (5.4 g L^−1^), sodium chloride (7 g L^−1^), pH 6.0–6.5). The platelet suspension was mixed with PGI_2_ (1:10000) vol:vol prior to centrifugation at 1200 *g* for 10 min. CGS buffer was removed and the platelet pellet was resuspended in Tyrode's buffer (sodium chloride (8.19 g L^−1^), potassium chloride (0.2 g L^−1^), sodium bicarbonate (1.01 g L^−1^), sodium phosphate monobasic (0.055 g L^−1^), glucose (0.991 g L^−1^), magnesium chloride (0.038 g L^−1^), calcium chloride (0.118 g L^−1^)) at 2.5 × 10^8^ platelets mL^−1^.

Despite the inclusion of different blood donors, the same response of PRP, EnP, and control media was repeatedly observed, further supporting the efficacy of PRP as a therapeutic agent.

*Cell Culture*: HUVECs—a commonly used pooled‐population of ECs whose lack of a blood antigen allows for the use of fresh donor blood without the need for donor‐matching—were used. HUVECs (Lonza) were cultured according to the manufacturer's guidelines using EBM Basal Medium supplemented with EGM Endothelial Cell Growth Medium SingleQuots Supplements (Lonza). For all experiments, HUVECs were used between passage 3 and 7. For 2D experiments, collagen‐coated coverglass was used as a substrate upon which HUVECs were cultured to confluency prior to perfusate treatment. In addition, human coronary artery smooth muscle cells (HCASMCs, Lonza CC‐2583) were cultured in SmGM‐2 smooth muscle cell growth medium (Lonza, CC‐3182). This cell type was transduced with pGIPZ NSC lentivirus overnight in SmGM‐2 supplemented with 10 ug mL^−1^ protamine sulfate (Sigma, P3369) to generate GFP‐SMCs. Purified GFP‐SMCs were then selected 2 days after transduction in SmGM‐2 supplemented with 2 ug mL^−1^ puromycin (InvivoGen, ant‐pr‐1) for 1 week. GFP‐SMCs were expanded in SmGM‐2 and used from P2‐4.

*Microvascular Network Fabrication*: Microvascular networks were made from 7.5 mg mL^−1^ collagen gel as described previously.[qv: 11b] Briefly, collagen was injected into a polyethyleneimine/glutaraldehyde‐treated Plexiglas housing top half that formed negative impression of a microvessel network along with a polydimethylsiloxane (PDMS) stamp. Inlet and outlet ports were formed by inserting stainless steel dowel pins before injecting the collagen. The Plexiglas bottom half was consisted of a flat layer of collagen compressed by a flat PDMS surface that was gelled on top of a standard coverslip. The collagen was allowed to gel for 30 min at 37 °C. After gelation, the PDMS pieces were removed and the two halves were brought together to seal the microvessel network. Culture medium was then added to the reservoirs and incubated for 2 h before cell seeding. To seed the vessels, 10 µL injections of HUVECs at a density of 8 × 10^6^ cells mL^−1^ were delivered to the inlet of an aspirated channel. Flow was allowed by replenishing media in the inlet reservoir twice per day for at least 3 days. Perfusates were then delivered via the inlet reservoir.

*Immunofluorescence Analysis*: Immunofluorescence images of cells on slides and within intact microvessels in situ were taken using a Nikon A1R Confocal Microscope (Nikon, Tokyo, Japan). Image stacks were acquired with a z‐step between successive optical slices of 2–3 µm. Cross‐sections, projections, and 3D reconstructions were generated from z‐stacks of images using ImageJ software.

In 2D and 3D experiments, vWF expression was quantified by setting a binary threshold on the vWF immunofluorescence channel. The area of vWF was corresponded to the number of pixels counted in the image using ImageJ. This number was divided by the total number of pixels in the region of interest and then, multiplied by 100 to give the percentage of vWF present in a given area of cells. Three images were analyzed for three separate devices. Data for each device were then averaged and used to calculate standard error of the mean (SEM) with *n* = 3.

*Ultrastructural Analysis*: Microfluidic vascular networks were examined under TEM as described previously.^17^ Briefly, networks were fixed by half‐strength Karnovsky's solution (2% paraformaldehyde/2.5% glutaraldehyde in 0.2 m cacodylate buffer) for 20 min followed by full immersion following disassembly in the same fixative solution for several days. Next, samples were post‐fixed with 2% OsO_4_ in 0.2 m cacodylate buffer, then dehydrated using immersions in graded solutions of ethanol, then propylene oxide (PO), before 1:1 PO/Epon 812 (Ted Pella Inc., Redding, CA) immersion overnight. Epon blocks were cured for 48 h at 60 °C then sectioned into ultrathin sections (70 nm) and placed onto grids. Grids were stained with uranyl acetate for 2 h and lead citrate for 5 min, then imaged using a JEOL JEM‐1400 Transmission Electron Microscope (JEOL Ltd., Japan) using a typical acceleration voltage around 100 kV. Images were acquired with a Gatan Ultrascan 1000XP camera (Gatan, Inc., Pleasanton, CA).

For 3D constructs, mitochondrial membrane potential and mass were quantified by confocal microscopy with a Nikon A1R microscope, using a 10x objective. Cells were stained with 30 × 10^−9^ m TMRM (Invitrogen) and 50 × 10^−9^ m MitoTracker Green (Invitrogen) for 30 min at 37 °C. To achieve baseline TMRM fluorescence, z‐stack images of the constructs were acquired every 60 s for 5 min. Mitochondria were depolarized using 20 m CCCP (Sigma‐Aldrich) and the resulting fluorescence value was subtracted from the baseline fluorescence to obtain the normalized mitochondrial membrane potential intensity.

*Permeability Assessment*: Perfusion of 70 kDa FITC‐dextran was driven by a hydrostatic pressure drop through the vessel. Using fluorescence microscopy with a 1 s sampling rate for 1 min, the distribution of fluorescence intensity during the transient flow of dextran was used to estimate the averaged apparent permeability of the kidney endothelium using Matlab processing in methods similar to Yuan et al.^18^ Fluorescence plot profiles were obtained using ImageJ software.

*RNA‐seq Data Analysis*: Total RNA was extracted from engineered microvessels by perfusing RLT buffer into the network at 250 µL min^−1^ for 1 min. Using an RNeasy kit (Qiagen), RNA was purified to reach 400 ng at concentration above 50 ng mL^−1^ for RNA‐seq. Base calling was performed using Illumina's Real‐Time Analysis software v1.18.66.3. Reads that failed to pass Illumina's base call quality filter were discarded, followed by using Cutadapt v1.11 to trim adapter sequence, discarding paired reads where either read in the pair had a length < 25. RNA‐seq samples were aligned to hg38 using Tophat.^19^ Gene‐level read counts were quantified using htseq‐count using Ensembl GRCh37 gene annotations.^20^ Genes with total expression above 10 normalized read counts summed across RNA‐seq samples were kept for further analysis. The princomp function from R was used for principal component analysis. DESeq was used for differential gene expression analysis with fold changes >1.5 and FDR < 0.1 as criteria to be considered differentially expressed.^21^ The topGO R package was used for GO enrichment analysis.^22^ Gene expression data set was then submitted to the public database in Gene Expression Omnibus.

*Whole‐Blood Perfusion*: Whole blood was drawn fresh and then fractionated by centrifugation at 200 *g* for 15 min. PRP was then separated and platelets were labeled by incubating with CD41a‐PE for 30 min at room temperature. Whole blood was then reconstituted and perfused at constant pressure for 5 min under live fluorescence imaging with a 1 s sampling rate.

*Two‐Photon Ablation Injury Model*: Vessel injury was performed by multiphoton photoablation using a Mai Tai DeepSee Ti:S laser (maximum power 2.57 W) coupled with an Olympus FV1000 MPE BX61 Microscope fitted with a water‐immersion objective lens (25x, NA = 1.05). Vessel regions were first identified for injury by conventional multiphoton imaging of constitutively expressing GFP‐expressing HUVECs and by second harmonic generation of collagen microfibers (λ_ex_ = 860 nm, λ_detector_ = 420–460 nm). Areas to be damaged were defined as 3D regions of interest using the Olympus Fluoview software then ablated by laser rastering performed at λ = 800 nm using 100% laser power, a pixel dwell time of 2 µs, and with 25–30 line repeat scans. Laser scanning in the *X*, *Y*, and *Z* dimensions was performed at ≈1 um step sizes. Cellular and collagen damage was confirmed by imaging identical areas after ablation using transmitted light, conventional multiphoton microscopy, or by second harmonic generation of collagen microfibers. Despite the presence of sodium citrate in the donor blood, there was the capability to induce coagulation in microvessels subjected to injury of 150 µm in length, suggesting that the lack of coagulation during PRP perfusion was not an artifact of this system.

*Statistical Analysis*: For all quantitative measurements, the entire population was used to calculate statistical significance, whereas mean values with *n* ≥ 3 groups were used to calculate standard error and graphical confidence intervals. Data were then analyzed in RStudio (Boston, MA) using a one‐way analysis of variance test, followed by post hoc analysis using Tukey‐HSD to determine significance between groups. On each graph, error bars represent ±2 SEM, a 95% confidence interval. A single asterisk was used for *p*‐values < 0.05, two asterisks for *p* < 0.01, three asterisks for *p* < 0.001, and four asterisks for *p* < 0.0001.

## Conflict of Interest

The authors declare no conflict of interest.

## Supporting information

Supplementary
