# Supplementary material for: Transforming Endothelium with Platelet‐Rich Plasma in Engineered Microvessels
Source: Adv Sci (Weinh). 2019 Oct 16;6(24):1901725. doi: 10.1002/advs.201901725 (PMC6918119; doi:10.1002/advs.201901725)
Supplement: Supplementary file 1 — Supplementary [file ADVS-6-1901725-s001.pdf]

Copyright WILEY-VCH Verlag GmbH & Co. KGaA, 69469 Weinheim, Germany, 2019.

Supporting Information

for *Adv. Sci.*, DOI: 10.1002/advs.201901725

Transforming Endothelium with Platelet-Rich Plasma in Engineered Microvessels

*Ryan J. Nagao, Raluca Marcu, Yuliang Wang, Lu Wang, Chris Arakawa, Cole DeForest, Junmei Chen, José A. López, and Ying Zheng**

Supplementary Materials

Transforming Endothelium with Platelet-rich Plasma in Engineered Microvessels

Nagao R.J.[1], Marcu R.[1], Wang Y.[2,3], Wang L.[1], Arakawa C.[1], DeForest C.[1,3,4], Chen J.[6], López J.A.[6,7], Zheng Y.[1,3]*

Departments of [1]Bioengineering, [2]Computer Science and Engineering, [3]Institute for Stem Cell and Regenerative Medicine, [4]Chemical Engineering, University of Washington, Seattle, Washington, 98195; [6]Bloodworks Northwest Research Institute, Seattle, Washington, 98102. [7]Department of Medicine, University of Washington, Seattle, Washington, 98109

* <u>Corresponding author:</u>

Ying Zheng, PhD., yingzy@uw.edu

SUPPLEMENTARY FIGURES:

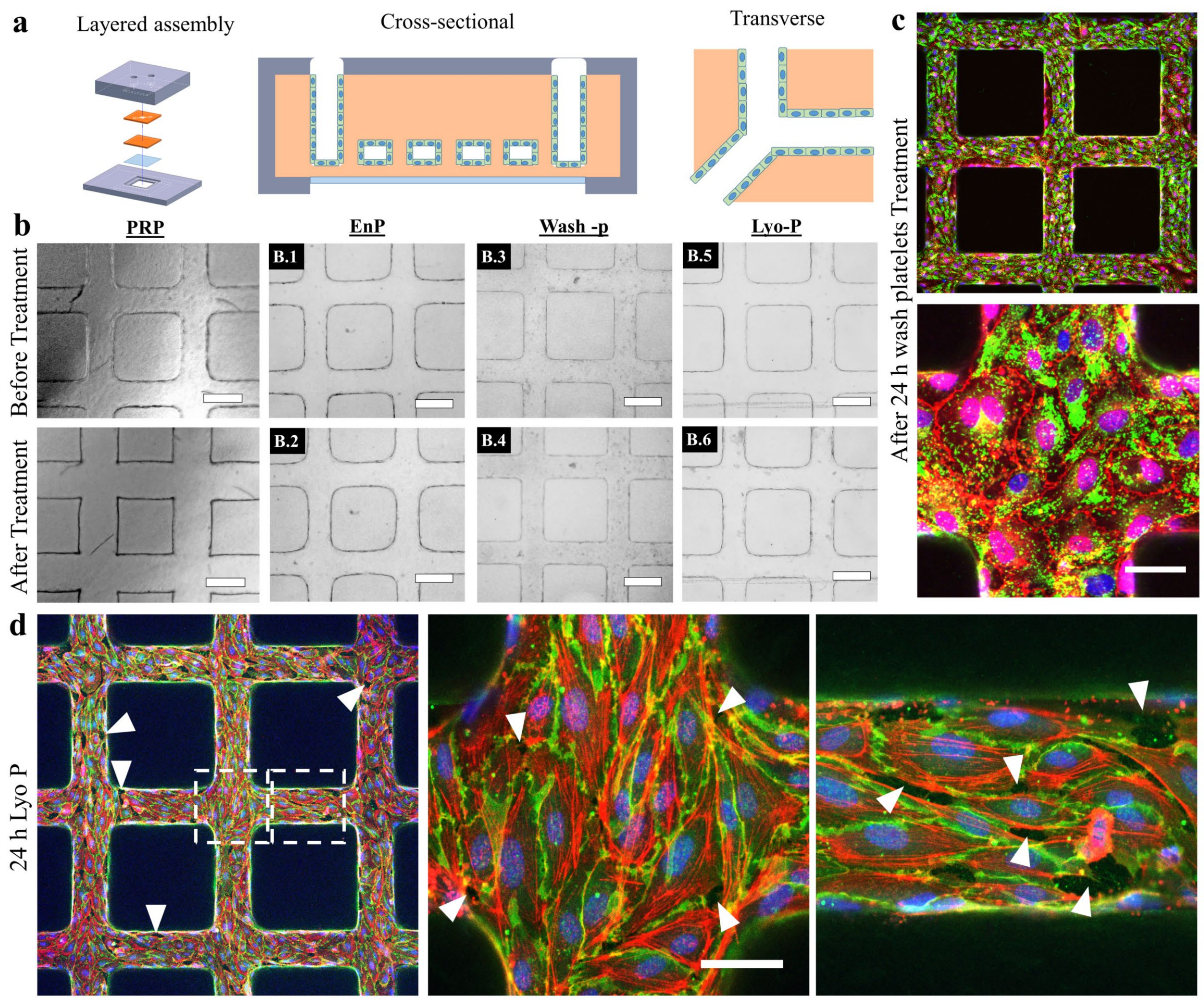

Supplementary Figure 1. PRP treatment uniquely transformed microvascular endothelium. **a**. Schematic of microvessel fabrication. **b**. Bright field images of microvessels before and after treatment with PRP, EnP, washed platelets (Wash-p) and lyophilized platelets resuspended in EnP (Lyo-P). **c**. Z-stack confocal image projections of microvessels after 24 h wash platelet treatments. Red: CD31, green: VWF, and blue: nuclei. **d**. Z-stack confocal image projection and zoomed view of vessel junction and branches of microvessels after 24 h Lyo P treatment. Arows: junctional defects or retracted regions of endothelium. Scale bars: **b**, 100 um; **c-d**, 50 um.

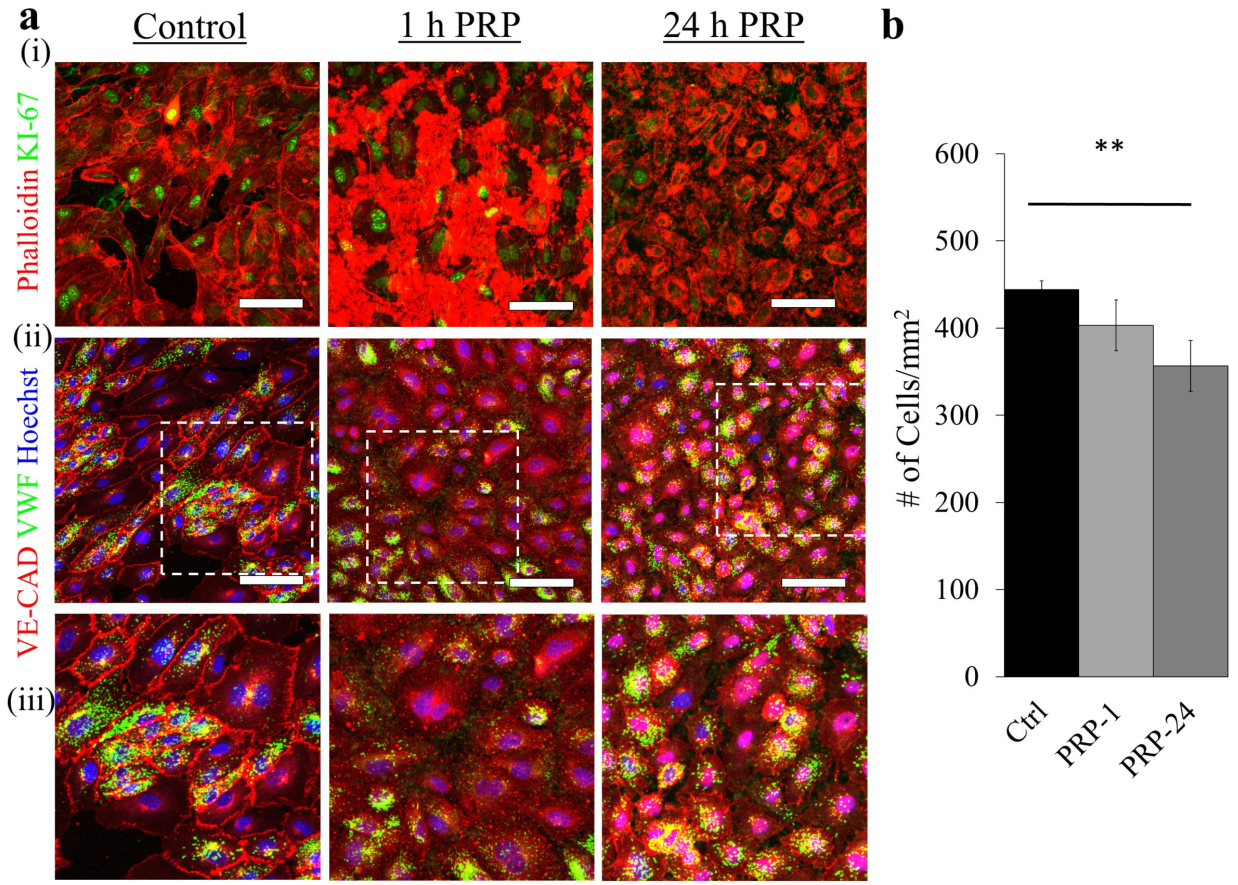

Supplementary Figure 2. 2-D PRP treatment lead to endothelial retraction. a. comparison of vessels in control, after 1 h PRP and 24 h PRP. (i) Red: F-actin, Green: Ki67. (ii-iii) Red: VE-Cad, green: VWF, and blue: nuclei. (iii) enlargements of dash boxes in (ii). Scale bar: 100 μm. **b**. Quantification of the number of cells per mm^2 for three conditions. **: $p < 0.01$.

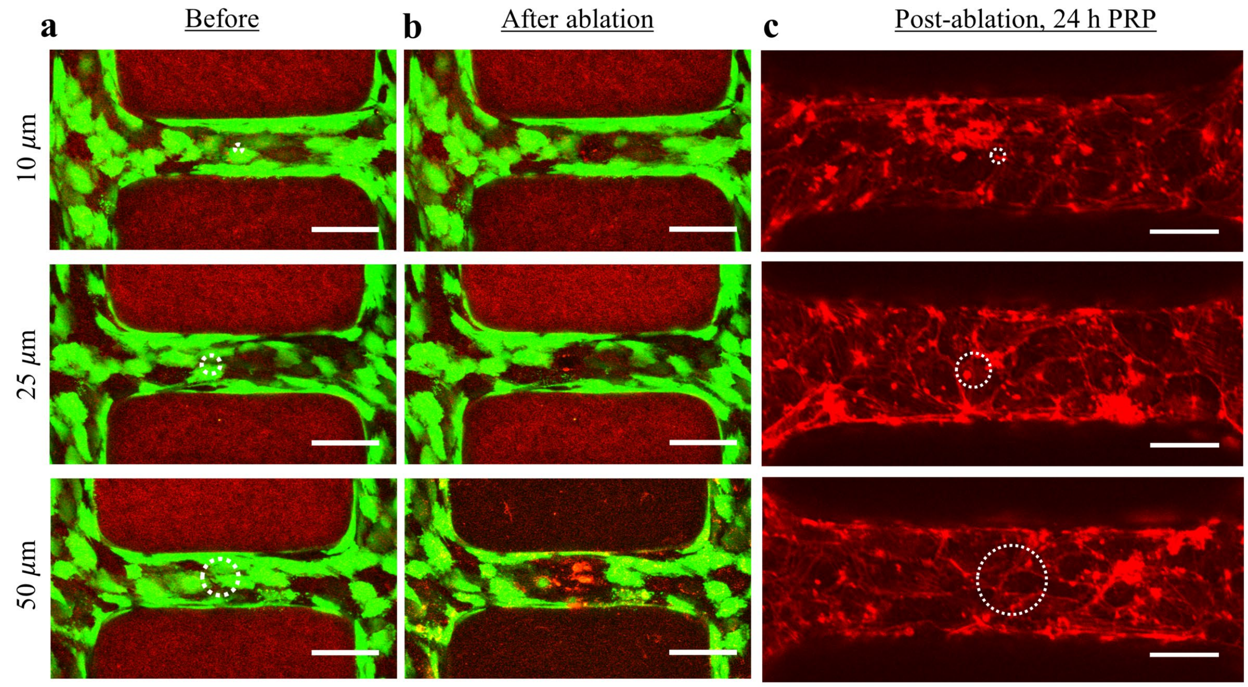

Supplementary Figure 3. Spotted vascular ablation led to full healing of endothelium after PRP treatment. Representative vessel branches (**a**) before, (**b**) after ablation, and (**c**) after 24 hr PRP perfusion post-ablation. Dash circles: ablation region with size of 10, 25, and 50 μm. Scale bars: **B**, 100 μm; **C**, 50 μm.

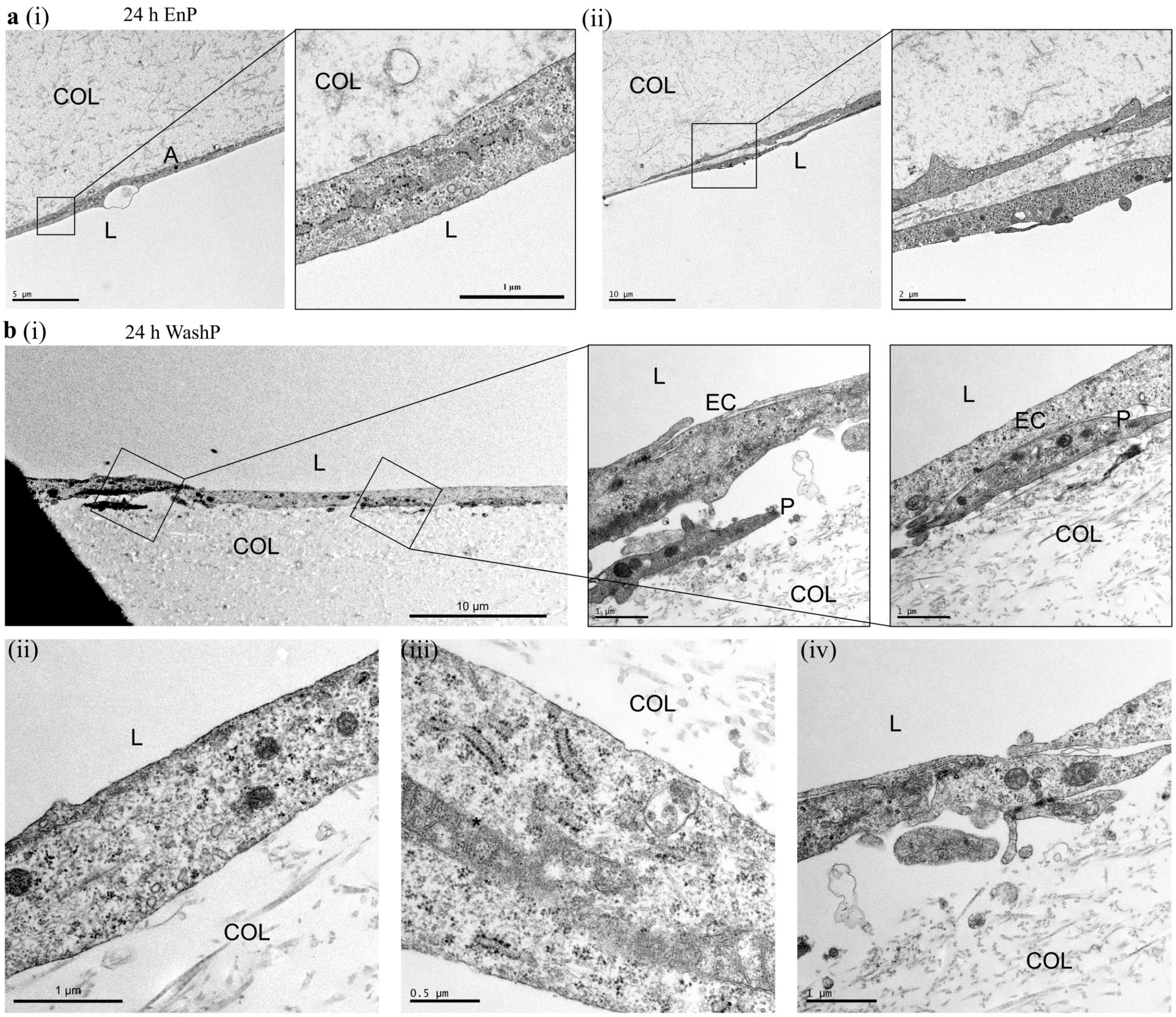

Supplementary Figure 4. Ultrastructural analysis of endothelium after 24 h treatment with enriched plasma and washed platelets. a. Transmission electron micrographs of vessels treated with enriched plasma. Increased protein material was seen on the abluminal side within the collagen matrix (i) and between overlapping endothelial cells (ii) suggesting the loss of serum protein. **b**. Washed platelets were found on the abluminal surface of endothelial cells and were partially activated. Changes to the submembranous actin web (ii) and mitochondria (iii)were consistent with PRP treatment. Junctions (iv) appeared normal.

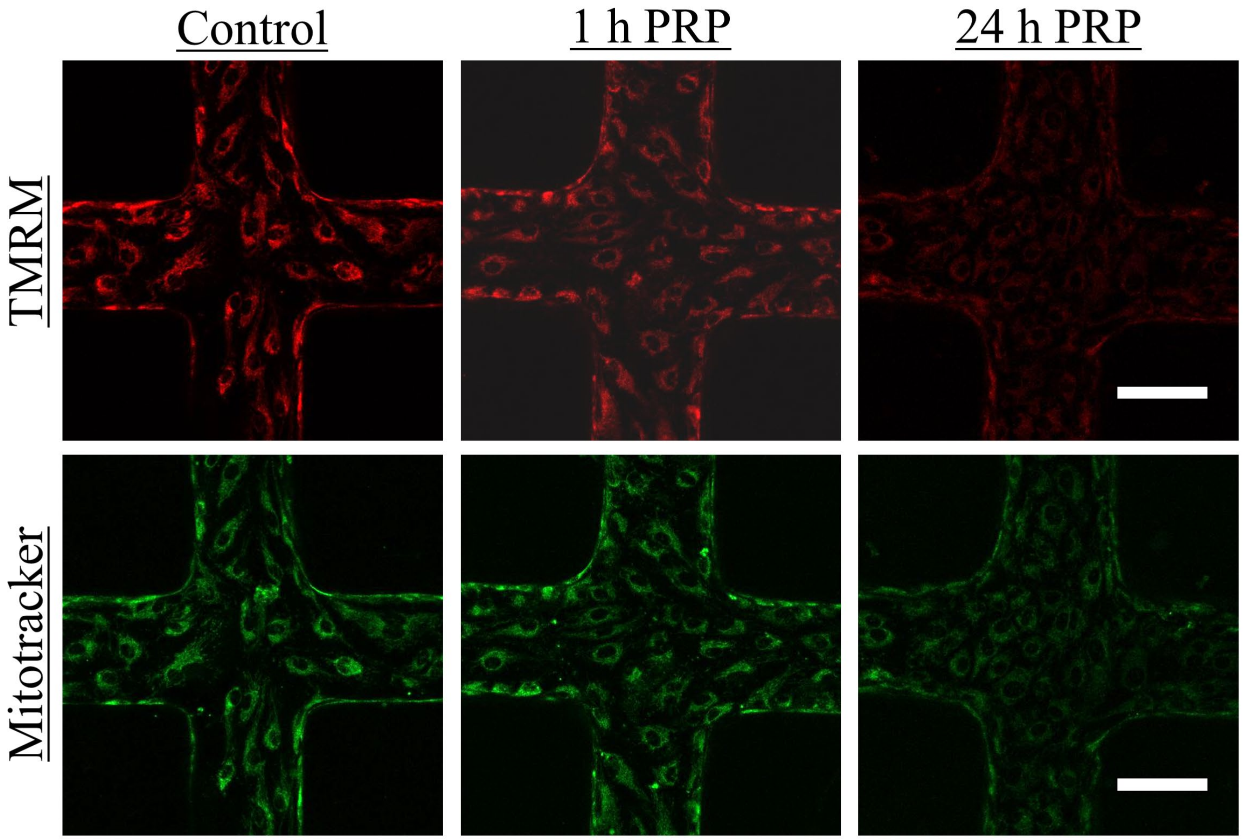

Supplementary Figure 5. Representative live image of microvessels with TMRM and MitoTracker Green staining in control, 1h PRP and 24 h PRP conditions. Scale bars: 100 μm.

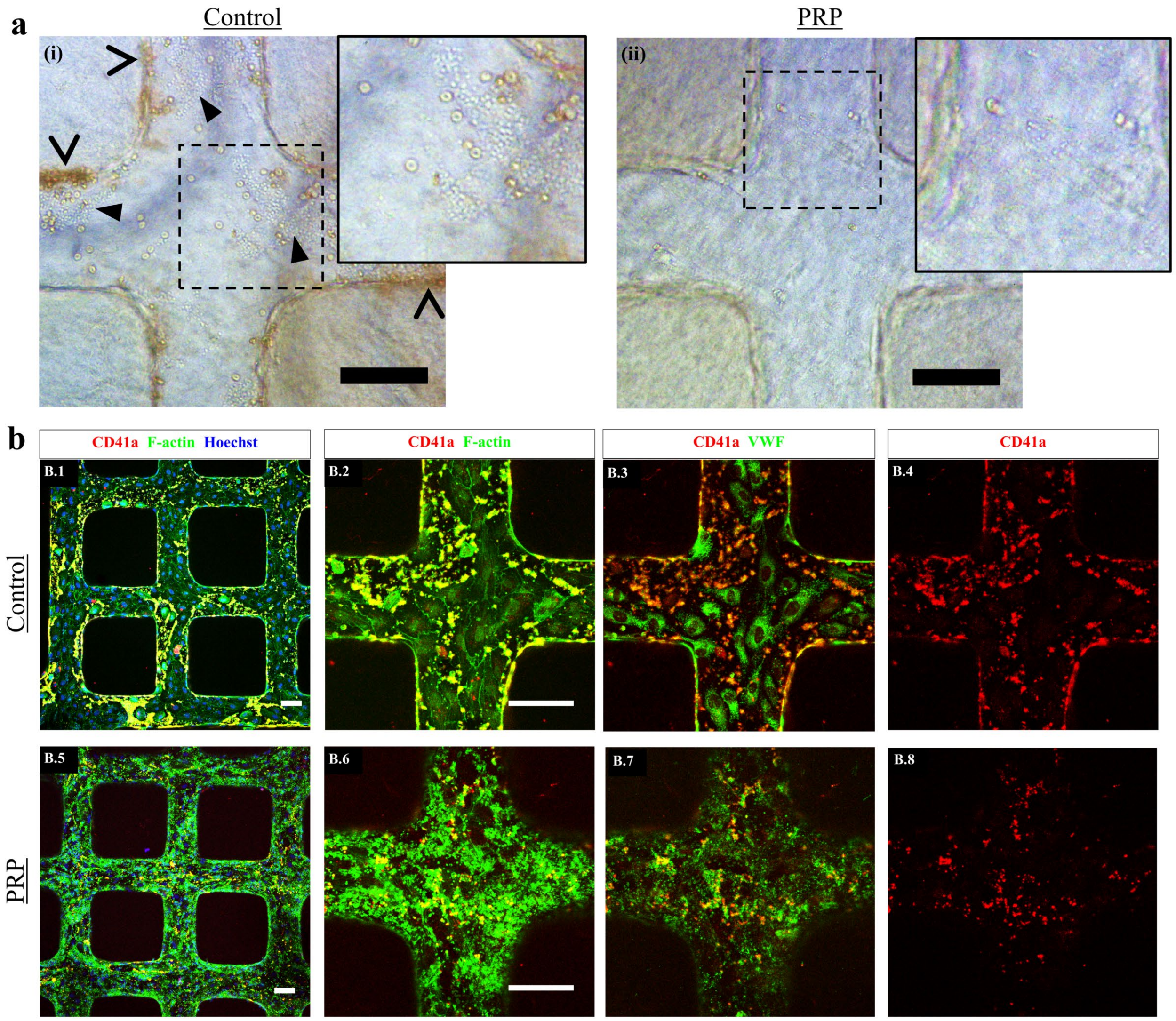

Supplementary Figure 6. PRP treatment lead to less thrombogenicity of the microvessel walls. a. brightfield images of control and PRP treated microvessels after whole blood perfusion and rinsing. **b**. Z-stack confocal fluorescence images of microvessels after whole blood perfusion. Red: CD41a and green: F-actin. Scale bars: 100 μm.
